# Towards a Lightweight Arabic Sign Language Translation System

**DOI:** 10.3390/s25175504

**Published:** 2025-09-04

**Authors:** Mohammed Algabri, Mohamed A. Mekhtiche, Mohamed A. Bencherif, Fahman Saeed

**Affiliations:** 1 Computer Science and Information Systems Department, College of Applied Sciences, AlMaarefa University, Riyadh 13713, Saudi Arabia; 2 King Salman Center for Disability Research, Riyadh 11614, Saudi Arabia; 3 Computer Engineering Department, College of Computer and Information Sciences, King Saud University, Riyadh 11543, Saudi Arabia; 4 Computer Science Department, College of Computer and Information Sciences, Imam Mohammad Ibn Saud Islamic University (IMSIU), Riyadh 11432, Saudi Arabia; faesaeed@imamu.edu.sa

**Keywords:** sign language translation, lightweight model, signer-dependent and signer-independent modes, attention mechanism

## Abstract

There is a pressing need to build a sign-to-text translation system to simplify communication between deaf and non-deaf people. This study investigates the building of a high-performance, lightweight sign language translation system suitable for real-time applications. Two Saudi Sign Language datasets are used for evaluation. We also investigate the effects of the number of signers and number of repetitions in sign language datasets. To this end, eight experiments are conducted in both signer-dependent and signer-independent modes. A comprehensive ablation study is presented to study the impacts of model components, network depth, and the size of the hidden dimension. The best accuracies achieved are 97.7% and 90.7% for the signer-dependent and signer-independent modes, respectively, using the KSU-SSL dataset. Similarly, the model achieves 98.38% and 96.22% for signer-dependent and signer-independent modes using the ArSL dataset.

## 1. Introduction

According to the World Health Organization (WHO), the number of people experiencing hearing loss is expected to reach to 2.5 billion by 2050 [1]. Over 700 million people of them will have disabling hearing loss; a subset of these uses sign language as a primary language. Based on the General Authority of Statistics in KSA, the number of people with a disability is 1,349,585, with 6.2% of them having hearing disability [2]. Hence, there is a pressing need to build a two-way sign language translation system to simplify communication and overcome barriers between deaf people and non-deaf people. In translating speech to sign language, many achievements have been met and work completed [3]. However, in the opposite direction, translating sign to text, achievements thus far are not at the same level. Current systems give high accuracy when training and testing from databases, but the accuracy is not acceptable for online translation. Moreover, current systems work on desktop computers, requiring high computation power, while building such a system on mobile phones would reduce the computation power. Moreover, building a high-performance lightweight sign language translation system that works in real time remains a challenging task [4,5]. Hence, this study aims to build a high-performance sign language translation system that can be deployed for real-time scenarios.

Saudi Sign Language (SSL) was the focus of this study, so two well-known SSL datasets were used. Both are word-level sign language datasets recorded as videos for each sign, including signs where the gesture has static hand shape. Accordingly, the proposed model is designed for a dynamic sign recognition system. Sign language datasets are important for building a high-performance translation system [4]. Due to the difficulty of engaging deaf people with dataset recording, most datasets rely on a limited number of signers, with each signer repeating the sign several time [5]; thus, in this study, we investigated the optimal number of signers and repetitions for building a highly accurate sign language translation system. To do so, several experiments were suggested in this study to study the effects of the number of signers and repetitions on system performance. Experiments were conducted in both signer-dependent and signer-independent modes. The signer-dependent mode was used to study the effects of the number of repetitions, while the signer-independent mode was used to study the effects of the number of signers in sign language datasets.

In signer-dependent mode, a system is trained and tested using data from the same signers, while in signer-independent mode, a system is trained using data from some signers and tested using data from other unseen signers. Signer-independent mode is more challenging than signer-dependent mode, and it is important for building real-world applications [6]. The main contributions of this paper are as follows: developing a lightweight dynamic sign language recognition system that works in real time; testing the performance of the model by conducting several experiments in both signer-dependent and signer-independent modes; and using two Saudi Sign Language datasets for evaluation.

The rest of this study is organized as follows: Section 2 reviews related work on sign language translation systems. Section 3 describes the datasets utilized in this study, while Section 4 details the feature extraction process applied to the selected datasets. The model architecture and training configuration are presented in Section 5, followed by the results and discussion in Section 6. An ablation study of the effects of model components and network architecture is conducted in Section 7. Finally, Section 8 provides the conclusions.

## 2. Related Studies

This section presents some related studies on sign language recognition systems, especially for Arabic sign language. Moreover, an overview of existing Arabic sign language datasets is provided. Sign language recognition systems are classified as vision-based and sensor-based systems. Our focus is on vision-based sign language translation systems [7].

An alphabet-level Arabic sign language translation system was proposed in [8]. A dataset of 9240 images derived from 28 Arabic alphabet characters was used. They obtained a 98.64% accuracy for hand detection, 99.5% for classification accuracy of all letters using KNN, and 97.5% for 14 Quranic dash letters. A deep learning-based CNN model was proposed for an Arabic alphabet sign translation system in [9]. They used the RGB AASL dataset [10], obtaining a 97.4% validation accuracy. A vision transformer was used for an Arabic sign language letter translation system in [11]. The ArSL2018 dataset, which consists of 54,049 images in 32 classes [12], was utilized. The proposed model achieved a 99.3% accuracy. A multi-layered CNN model was proposed in [13] and tested using the same ArSL2018 corpus, achieving a 99.7% accuracy. A YOLO-based model was used for an Arabic sign language letter translation system in [14]. The authors used the ArSL21L dataset [15], which consists of 14,202 images of 32 unique letters, collected from 50 signers. They achieved a 99% detection accuracy.

A word-level Arabic sign language translation system was proposed in [16]. A dataset of 30 words was used, and a Euclidean distance classifier obtained a recognition accuracy at 97% in signer-independent mode. Deep learning models were used for a word-level sign language translation system proposed in [17], where a corpus of 7030 images representing 14 Arabic words was developed to evaluate the models. Diverse CNN architectures such as VGG, MobileNet, DenseNet, and Inception were utilized, and the best accuracy was achieved by VGG16 at 98.65%. A word-level signer-independent Arabic sign language translation system was proposed in [18]. A dataset of 23 isolated Arabic words [19], developed by three signers, was used to evaluate the proposed model. They achieved a 89.5% accuracy by using DeepLabv3+ for performing hand segmentation. An Arabic sign language dataset (ArSL) of 80 signs was developed in [20], with each sign performed by 40 signers, with five repetitions of each sign. They proposed using 1D-CNN with skeleton data and obtained accuracies of 89.62% and 88.09% for signer-dependent and signer-independent modes. A 3D CNN model was proposed in [21] for 40 dynamic signs from the ArSL dataset, achieving accuracies of 98.12% and 84.38% for signer-dependent and signer-independent modes, respectively. The same authors also proposed another system in [22], using 3DNN with MLP fusion and an auto-encoder, and obtained accuracies of 98.75% for signer-dependent mode using auto-encoder fusion and 87.69% for signer-independent mode utilizing MLP fusion. A 3D-GCN model with an attention mechanism was proposed in [23] for a sign recognition system. A large Arabic sign language dataset, called KSU-SSL, consisting of 293 signs performed by 32 signers, was utilized. The authors obtained an accuracy of 97.25% in signer-dependent mode.

A sentence-level Arabic sign language translation system was proposed in [24] using LSTM. The ArabicSign dataset [25], which consists of 50 sentences with 9335 samples, was used. The proposed model obtained a test accuracy of 88.75%. Another sentence-level study was proposed in [26], whose authors used a dataset of 40 sentences performed by only one signer. The proposed model used biLSTM layers and obtained a sentence-level accuracy of 92.6%. An Arabic sign language dataset consisting of 30 common sentences, performed by only three signers, was developed by [27]. They proposed a model based on a temporal convolutional network (TCN) and obtained an accuracy of 99.5%.

For other sign languages, ref. [4] provides a comprehensive review of sign datasets and techniques for sign language recognition.

## 3. Sign Language Datasets

In this study, two well-known Arabic sign language datasets, KSU-SSL and ArSL, were used to evaluate the proposed system. Table 1 presents a summary of dataset statistics. KSU-SSL [28] was used to evaluate the proposed system, while ArSL [20] was used for comparing the performance of our proposed system with state-of-the-art research. KSU-SSL was designed for the Saudi Sign Language and consists of 293 signs from 10 domains. The healthcare domain is predominant, with 133 signs, representing about 45% of the total signs. The rest of the signs were selected from daily life signs. All signs were performed by 33 signers, with each signer repeating the signs four times; a fifth performance was noted, as signers wore gloves in an early recording before later switching to painted hands. All signs were performed according to the Saudi Sign Language dictionary [29] developed by the Saudi Association for Hearing Impairment. The data were recorded in RGB, IR, and mobile modalities. In this study, we did not use the sample with colored hands and considered only the remaining four RGB repetitions. We could not find the videos of signer 33, hence we used only the videos of 32 signers. ArSL [20] consists of 80 signs performed by 40 signers. Each signer performed each sign five times. They used different recording devices such as a Kinect v1, Kinect v2, and Sony HandyCam. The data were recorded in RGB, depth, and skeleton modalities. In this study, we used only the RGB modality from both datasets.

**Table 1:** Summary of dataset statistics.

Dataset	Number of Signers	Number of Signs	Number of Repetitions	Total Number of Samples
KSU-SSL [28]	33	293	5 (one with colored finger)	145,035
ArSL [20]	40	80	5	16,000

## 4. Feature Extraction Step

We used the MediaPipe framework [30] to extract the landmarks from the frames of videos. For each frame, 95 hands and pose landmarks were extracted, as presented in Table 2. Each landmark was represented by (x, y, z) coordinates. For each hand, the following landmarks or features were extracted: 21 basic landmarks, 5 position landmarks between the fingers and the wrist, 4 angles landmarks between adjacent fingers, and 1 curl landmark for each finger. The four position landmarks between the fingers and wrist were used to provide a translation invariant to allow the model to recognize signs in different locations. The four angles between the adjacent finger features were used to provide rotation invariance by capturing the relationships between fingers. The curl landmarks for each finger were useful for distinguishing the digit signs. Additionally, 25 pose landmarks were extracted as follows: 21 from upper body landmarks, 2 position features to recognize the spatial relationship between the hands and face, and 2 velocity features to provide temporal features mainly for dynamic signs.

## 5. Model Architecture

**Figure 1:**
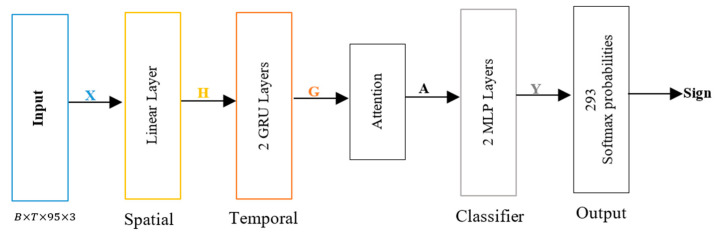
Model architecture for Arabic sign language recognition, *X*: input, *H*: spatial feature, *G*: temporal feature, *A*: weighted features, and *Y*: output.

The investigated model consists of four main parts (as shown in Figure 1): spatial encoding, temporal encoding, an attention mechanism, and a classifier. This model combines spatial-temporal attention techniques while ensuring CPU-friendly operation to allow real-time applications. We assume the input is a sign language video, which is fed to MediaPipe to extract features represented by X, where $X\in R^{B(Batch)\times T(Frames)\times N\times D}$, N represents the number of landmarks (95), and D represents the (x, y, z) coordinates of each landmark. For spatial feature extraction, a linear layer, followed by an ReLU activation function (σ), is used to find $H=\;\sigma (X_{t}W+b)$, where $H\in R^{B\times T\times F}$ and F is the feature dimension. Then, the spatial features H are fed to the two bidirectional GRU layers to capture the temporal features, and the output of forward and backward GRU is represented by $G\in R^{B\times T\times 2H}$, where G is the hidden dimension. Then, the attention layer is used to calculate the weighted features $A\in R^{B\times 2H}$ from G as $A=\;\sum_{t=1}^{T}\alpha_{t}G_{t}$, where α is the attention weights. Finally, the classifier head applies the linear projection to produce the output $Y\in R^{B\times K}$, where $Y=AW_{c}+b_{c}$, K is the number of sign classes, and the final classification output is obtained as $softmax\left(Y\right)$.

### Model Training

Eight experiments were investigated in this study using the KSU-SSL dataset. These experiments were conducted to study the effects of the number of signers and repetitions for each sign in both signer-dependent and signer-independent modes. Our goal in these experiments was to find the minimum number of signers and repetitions needed to achieve a satisfactory performance for the sign recognition system. In KSU-SSL, each signer performed four repetitions for all signs; thus, in signer-dependent mode, we used the first, first and second, and first, second, and third repetitions for training in Exp_1, Exp_2, and Exp_3, respectively. For all three experiments, the fourth repetition was used for testing, and 10% of the training samples were used for validation. For signer-independent mode, signers 26–32 were used for testing in all experiments, while for training, we used only five signers in Exp_4, then another five signers were added to the training samples for each experiment up to Exp_8. In Exp_4 to Exp_8, 10% of training samples were used for validation. Table 3 summarizes the data split of all experiments conducted on the KSU-SSL dataset.

The following training hyperparameters were used for all experiments: the number of training epochs was 100, the batch size was 32, the learning rate was 0.001, and the optimizer was the Adam optimizer. In signer-independent mode, the Exp_8 model achieved the best validation accuracy of 97%, while the training loss decreased from 3.47 to 0.014 and the final validation loss was 0.118.

## 6. Results and Discussion

In this section, the results of the conducted experiments for evaluating the proposed model are presented. The system accuracy is presented for both signer-dependent and signer-independent modes. Then, the accuracy per signer in both modes is reported. The evaluation of the model in a real-time scenario is also conducted. Finally, representative examples of testing samples are provided.

Table 4 shows the accuracies of all experiments. For signer-dependent mode, we obtained an accuracy of 92.9% by training the system using only one repetition, which provided very good results considering that we only used one sample per each of the 25 signers in the training samples. When we used two and three repetitions per signer, the accuracy increased to 96.4% and 97.7%, respectively, corresponding to 3.5% and 1.3% improvements.

On the other hand, for the effect of the number of signers in signer-independent mode, when training the system using only five signers, the accuracy obtained was only 46.7%, increasing to 60.3% by increasing the number of signers to ten. Upon increasing the number of signers to 15, the accuracy increased to 84.7%. Moreover, upon increasing the number of signers to 20 and 25, the accuracy slightly increased to 87% and 90.7%, respectively. From this result for signer-independent mode, we can conclude that the number of signers plays a vital role in the performance of sign translation systems and, therefore, there may be a minimum number of signers required by the database to develop a high-performance system.

**Figure 2:**
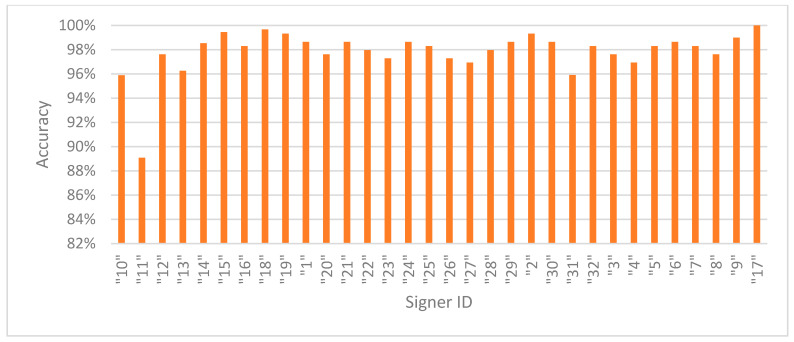
Accuracy per signer in signer-dependent mode (Exp_3).

**Figure 3:**
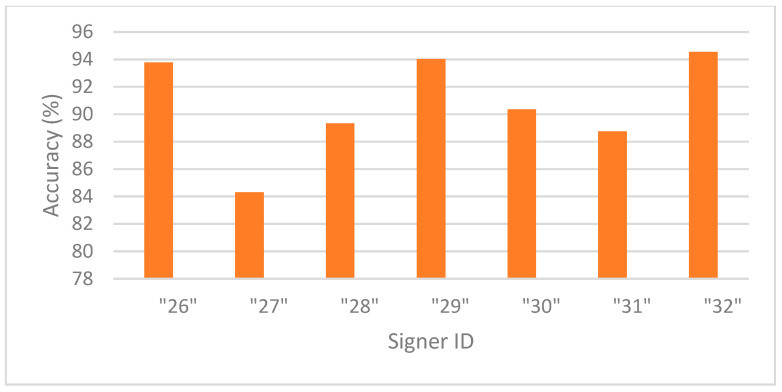
Accuracy per signer in signer-independent mode (Exp_8).

Figure 2 presents the accuracies in signer-dependent mode for each signer in Exp_3. Signer 11 is an outlier with an accuracy of 89%, while the rest of the signers have accuracies of almost 96% and above, indicating that the model is robust, effective, and well trained using signer-specific data. Figure 3 presents the accuracy in signer-independent mode for each signer at the testing stage using the model for Exp_8. All signer accuracies are above 88% except for signer 27, who has the lowest accuracy at 84.3%. These results indicate that the model generalizes well across unseen signers and learns well in a signer-invariant manner.

**Figure 4:**
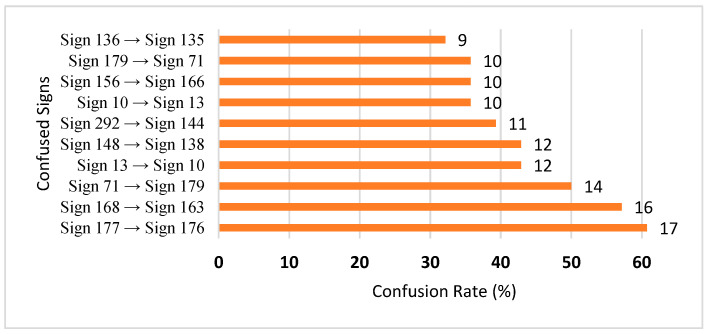
Top 10 confused signs in signer-independent mode (Exp_8).

The top 10 most confused signs for the Exp_8 model are presented in Figure 4. From Figure 4, the two highest confusions occur between sign 177 (menstruation) and sign 176 (menstrual_period), and sign 168 (letter_zei) and sign 163 (letter_taa). For the signs 177 and 176, we found that they were actually the same videos but with different labels. For the signs 168 and 163, the two signs have very similar motion performed with two fingers of the right hand.

### 6.1. Qualitative Analysis of Testing Samples

Table 5 shows a qualitative analysis of samples from the testing set using the model for Exp_8 in signer-independent mode. It presents the input frames, extracted landmarks, and model prediction. The first example is for the sign “five” performed by signer 32. The extracted landmark features originate only from one hand; in this case, the feature vector is padded with zeros to match the model’s input dimensions. The prediction matches the ground truth with a confidence score of 98.3%, demonstrating the robustness of the model for the signs of one hand. The second example is for the sign “family” performed by signer 29. This sign is performed using two hands, clearly reflected in the extracted landmarks. The model accurately predicts the sign with a high confidence score of 97%, showing the robustness of the model for the two-handed signs. The third example shows the robustness of the model in handling signs involving body movement, for example, when the head is turned to the left or right and the body is not directly facing the camera. Nevertheless, the model predicts the sign accurately with a confidence of 93.5%.

### 6.2. Real-Time Performance and Model Stability

This section aims to evaluate the capability of the model for performing real-time sign language recognition on low-resource devices by analyzing the runtime performance of the proposed model on both a CPU (Intel Core i7) and Raspberry Pi 5. Table 6 presents a comparison of the inference time performance when the model is executed on an Intel Core i7 versus when the model is executed on a Raspberry Pi 5 using the single sample latency, and frames per second (FPS). The metrics highlight the model’s capability to work in real-time and low-resource environments. The model size is 2.43 MB and the total number of training parameters is 635,174, showing that it has a lightweight architecture suitable to deploy on low-resource devices. The low latencies in the CPU and Raspberry Pi, which are 11.97 ms and 38.36 ms, respectively, as well as the estimated FPS of 83.6 in the CPU, indicate that the model supports real-time performance.

To evaluate the stability of the model under real-world conditions, we simulated such conditions by augmenting the MediaPipe key-points features. We applied three types of augmentations: uneven lighting, diverse background, and moving camera simulation. The evaluation was conducting using the Exp_8 model. Under uneven lighting, the accuracy decreased from 90.7% to 89.1%. With diverse background simulation, accuracy decreased to 84.5%. Under the moving camera simulation, the accuracy dropped to 84.7%. These results indicate that the model is stable and robust under uncontrolled conditions.

To evaluate model consistency, we trained the Exp_3 model with three different seeds. The model achieved an accuracy of 97.5% ± 0.05, with a 95% confidence interval of [97.39%, 97.65%]. Similarly, the Exp_8 model was trained with three different seeds and achieved an accuracy of 90.6 ± 0.54 with a 95% confidence interval of [89.24%, 91.94%].

### 6.3. Comparing the Model’s Performance with Other Models

To compare the model’s performance with state-of-the-art models, we evaluate the model using the ArSL dataset. ArSL consists of 80 signs signed by 40 signers, where each signer repeats the sign five times. We use the same data distribution used in [20] and compare our data with their results; thus, in signer-dependent mode, three repetitions of all signs are used for training, while the fourth and fifth repetitions are used for testing and validation, respectively. On the other hand, in signer-independent mode, 23 signers are used for training, 8 for validation, and 8 for testing. In Table 7, we can see that the proposed model obtains an accuracy of 98.38%, while the accuracy of [20] is 89.62% in signer-dependent mode. For signer-independent mode, our model achieves a 96.22% accuracy, while the accuracy of [20] is 88.09%. Thus, we can conclude that our model outperforms the model of [20] in both signer-dependent and signer-independent modes.

## 7. Ablation Study

In this section, an ablation study is conducted to examine the effects of the model components, hidden dimensions, and network depth on the performance of sign language recognition. We conduct the ablation study on the KSU-SSL dataset in signer-independent mode, as it is more challenging than signer-dependent mode. The baseline model architecture is described in Section 5.

Table 8 shows the ablation results of the model architecture components, obtained by removing the spatial, temporal, or attention modules from the baseline model. It also includes the results derived from replacing the GRU with an LSTM in the temporal complement and using a unidirectional instead of bidirectional variant.

From the obtained results, it is clear that the temporal module significantly impacts the model performance, as removing it reduced the accuracy to 81.5%. The spatial module also contributes to the baseline model, with the accuracy decreasing to 88.5% when it was removed. Replacing the GRU with the LSTM had minimal impact on accuracy, but it increased the number of parameters from 635,174 to 800,038. Hence, we used the GRU in the baseline model architecture. Using a unidirectional variant reduced the number of trainable parameters by up to 50% while maintaining high accuracy.

Table 9 shows the impact of the hidden dimensions of the model. In the baseline model, we used 128 dimensions; when this figure was increased to 256, the obtained accuracy increased to 91.1%, while the total number of parameters sharply increased to more than 2 M. In contrast, when it was decreased to 64%, the obtained accuracy decreased from 90.7% to 88.2% and the training parameters decreased from 635,174 to 178,470.

Table 10 shows the results for an ablation study on network depth. When using one GRU layer in the temporal part instead of two layers, the accuracy slightly decreases to 89.8%, while the total number of parameters decreases to 338,726. When the number of GRU layers increases to three, the total number of parameters increases to 931,622, while the accuracy remains nearly the same as that of the baseline. For the spatial part, increasing its depth to two layers slightly improves both the accuracy and the number of training parameters. Notably, using a single layer in the classifier part enhances the accuracy from 90.7% to 91.2%, with only a slight increase in training parameters, which may mean that this model is more suitable for real-time applications.

## 8. Conclusions

A high-performance lightweight sign language recognition model is presented and evaluated in this study. The model is evaluated using two well-known Saudi Sign Language datasets in both signer-dependent and signer-independent modes. Several experiments were conducted to study the effects of the number of signers and repetitions on sign language datasets. Through experiments, we found that the number of signers has a more significant effect on the model’s accuracy than the number of repetitions. Several ablation studies were conducted to study the effects of each part of the model. The model was evaluated for real-time application using several performance metrics such as FPS, latency, and model size. For future studies, we plan to evaluate the model using other sign datasets.

## Figures and Tables

**Table 2:** Summary of extracted landmarks.

Type	Landmarks
Each Hand	35 (21 hand landmarks + 5 position features + 4 angle features + 5 curl features)
Pose	25 (21 upper body landmarks + 2 position features + 2 velocity features)

**Table 3:** Data split for all experiments.

Training Mode	Experiment ID	Training Set	Validation Set	Testing Set
Signer-dependent mode	Exp_1	All signers, repetitions: 1	10% from the training set	All signers, repetitions: 4
	Exp_2	All signers, repetitions: 1, 2		
	Exp_3	All signers, repetitions: 1, 2, 3		
Signer-Independent mode	Exp_4	All repetitions of signers 1–5		All repetitions of signers 26–32
	Exp_5	All repetitions of signers 1–10		
	Exp_6	All repetitions of signers 1–15		
	Exp_7	All repetitions of signers 1–20		
	Exp_8	All repetitions of signers 1–25		

**Table 4:** Performance metrics for all experiments in signer-dependent and signer-independent modes (S: signer; R: repetition).

Mode	Experiment ID	Size of Training Data	Accuracy
Signer-dependent mode *effect of number of repetitions*	Exp_1	All S, one R	92.9%
	Exp_2	All S, two R	96.4%
	Exp_3	All S, three R	97.7%
Signer-independent mode *effect of number of signers*	Exp_4	5 S, three R	46.7%
	Exp_5	10 S, three R	60.3%
	Exp_6	15 S, three R	84.7%
	Exp_7	20 S, three R	87.0%
	Exp_8	25 S, three R	90.7%

**Table 5:** Examples of testing samples using Exp_8.

Signer ID	One Frame from the Input Video	The Extracted Features	Prediction Results
32	** 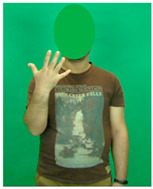 **	** 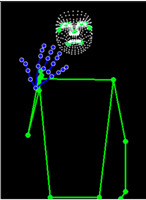 **	Class: *Five* Confidence: 98.3% Sequence length: 12 frames
29	** 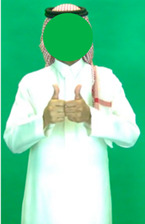 **	** 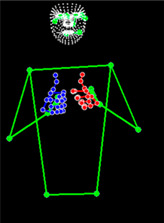 **	Class: *Family* Confidence: 97.0% Sequence length: 10 frames
28	** 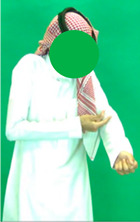 **	** 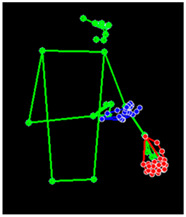 **	Class: *Injection* Confidence: 93.5% Sequence length: 12 frames

**Table 6:** Performance evaluation of the real-time scenario.

Performance Criteria	Value
Model Size	2.43 MB
Training Parameters	635,174
Single Sample Latency (Intel Core i7)	11.97 ms
Estimated FPS (Intel Core i7)	83.6
Single Sample Latency (Raspberry Pi 5)	38.36 ms
Estimated FPS (Raspberry Pi 5)	26.1

**Table 7:** Performance of the training modes on the ArSL dataset.

Training Mode	Experiment ID	Training Set	Validation Set	Testing Set	Accuracy	Accuracy [20]
Signer-dependent mode	Exp_9	All signers, repetitions 1,2,3	All signers, repetition 5	All signers, repetition 4	98.38%	89.62%
Signer-independent mode	Exp_10	Signers 1, 2, 10–31	Signers 3–9, 40	32–39	96.22%	88.09%

**Table 8:** Architecture component ablations.

Model	Test Accuracy	Total Number of Parameters
Baseline	90.7%	635,174
Baseline (no spatial)	88.5%	718,886
Baseline (no temporal)	81.5%	91,173
Baseline (no attention)	90.4%	602,149
Baseline (LSTM temporal instead of GRU)	90.1%	800,038
Baseline (GRU unidirectional)	89%	305,958

**Table 9:** Hidden dimension ablations.

Model	Test Accuracy	Total Number of Parameters
**Baseline**	**90.7%**	**635,174**
**Baseline (256 hidden dimensions)**	**91.1%**	2,384,166
**Baseline (64 hidden dimensions)**	88.2%	178,470

**Table 10:** Network depth ablations.

Model	Test Accuracy	Total Number of Parameters
Baseline	90.7%	635,174
Baseline (1 GRU layer)	89.8%	338,726
Baseline (3 GRU layers)	90.2%	931,622
Baseline (2 spatial layers)	90.8%	651,942
Baseline (1 classifier layer)	91.2%	639,782

## Data Availability

No new data were created or analyzed in this study.
